# Grape Pomace Ingestion by Dry Cows Does Not Affect the Colostrum Nutrient and Fatty Acid Composition

**DOI:** 10.3390/ani11061633

**Published:** 2021-05-31

**Authors:** Michal Rolinec, Daniel Bíro, Milan Šimko, Miroslav Juráček, Ondrej Hanušovský, Zuzana Schubertová, Lucie Chadimová, Branislav Gálik

**Affiliations:** 1 Department of Animal Nutrition, Slovak University of Agriculture in Nitra, Trieda A. Hlinku 2, 94976 Nitra, Slovakia; daniel.biro@uniag.sk (D.B.); milan.simko@uniag.sk (M.Š.); miroslav.juracek@uniag.sk (M.J.); branislav.galik@uniag.sk (B.G.); 2 Institute of Biodiversity Conservation and Biosafety, Slovak University of Agriculture in Nitra, Trieda A. Hlinku 2, 94976 Nitra, Slovakia; zuzana.schubertova@uniag.sk; 3 PD Kozárovce, 93522 Kozárovce, Slovakia; xmierna@is.uniag.sk

**Keywords:** wine by-product, bovine colostrum, fat quality

## Abstract

**Simple Summary:**

The introduction of alternative feedstuff such as grape pomace into the diets of dry cows could decrease the cereal needs in ruminant feeding systems and could modify the composition of colostrum. Grape pomace is a by-product of the wine industry. It contains polyphenols and fatty acids, which have the potential to improve the animal product quality. The nutritional quality of colostrum and the quality of colostral fat is affected by dry cow nutrition in the late stages of pregnancy. This study determined the potential of grape pomace feeding to increase colostral protein, which, according to the literature, is connected with the concentration of immunoglobulin G, better passive immunisation, and the health of calves. The addition of grape pomace was determined to have no effect on colostral nutrient and fatty acid concentrations. Thus, grape pomace can be used as a nutrient source for dry cows.

**Abstract:**

The utilisation of different by-products from the food industry as nutrient sources for farm animals is both possible and beneficial. Grape pomace is a by-product that contains polyphenols and fatty acids, both of which have the potential to improve the nutritional quality of cow colostrum. This study aimed to explore how the addition of grape pomace to the diet of dry cows affects the concentration of nutrients and fatty acids of colostrum. Sixteen Slovak spotted cows in late pregnancy were used in this study. From the seventh day before expected calving to the day of calving, cows in the grape pomace group received a diet supplemented with dried grape pomace, at 0.116 kg/cow/day. Colostrum samples were analysed for basal nutrients and fatty acid concentrations. Between the control and experimental groups, the nutrient and fatty acid concentrations of all the colostrum samples did not show significant differences. The nutrient levels in the colostrum from both groups of cows were typical, as related to the time from calving. The addition of the grape pomace into the diet of dry cows had no effect on nutrient concentrations and the fatty acid composition of the colostrum. The somatic cell score of the colostrum sampled at the 12th hour after calving (4.2 versus 2.6) was positively affected by grape pomace addition. The results of this study revealed that grape pomace (fed in an amount of 0.116 kg/cow/day) had no positive or negative effect on the base nutrients and fatty acids of cow colostrum, and, therefore, grape pomace can be used as a nutrient source for dry cows in small amounts.

## 1. Introduction

The ingestion of sufficient amounts of colostrum is essential for newborn calves. Colostrum is secreted by the udder from calving until a few days after calving. At first, colostrum provides energy and passive immunisation to newborn calves. Moreover, the fat and fatty acids play an important role in health and performance. Other colostrum components, such as lactose, minerals, vitamins, and biologically active substances, make the colostrum a complete feed for newborn calves. As stated by Mechor et al. [1], colostral protein concentration has a positive correlation with immunoglobulin G concentration. Therefore, the concentration of colostrum crude protein has an essential effect on the passive immunisation of newborn calves. Contarini et al. [2] reported that one of the least considered colostrum components is fat. Studies on colostrum fat mainly focus on its contents during the first days after calving. Nevertheless, the positive health effects of colostrum fat are promoted by oleic acid and various polyunsaturated fatty acids (PUFA) such as omega-3 fatty acids (n-3 FA) [3]. Certain PUFA such as n-3 FA play important roles for newborn calves, supporting the development of the emerging immune system and the brain and retina [4,5]. Considering the importance of colostrum for calf nutrition, the composition and quality of its fat fraction cannot be ignored and needs to be brought up to date [2].

Grape pomace is a wine by-product. It is currently used as feed because it is rich in several phytochemicals that have positive health effects [6]. Scuderi et al. [7] reported that grape pomace is rich in secondary compounds, including condensed tannins, and is used as a supplement in livestock feeding practices. Ianni and Martino [8] stated that grape pomace is a feed that is enriched by polyphenols and dietary fibre and has the ability to improve the quality of cow milk. First, a general increase in the concentration of PUFA was reported. Another study published by Ianni et al. [9] concluded that a diet supplemented with dried grape pomace did not affect the milk composition, but induced modifications in the fatty acid profiles in the milk. Castellani et al. [10] concluded that grape pomace as a dietary supplement in dairy cows may enhance milk fatty acids. The dietary inclusion of polyphenols decreased the saturated fatty acids (SFA) and increased the monounsaturated fatty acids (MUFA) and PUFA in milk [11]. Furthermore, Chedea et al. [12] fed dairy cows a diet containing dried grape pomace and reported higher concentrations of lactose in the milk. However, there is lack of information regarding the effect of grape pomace intake by dry cows on the nutritional composition of colostrum. Furthermore, the addition of grape pomace into the diet of dry cows can reduce the proportion of cereals in the diets of this category of cattle.

As a result of the reported effect on cow milk and the lack of information concerning the effect of grape pomace feeding on the fatty acid and nutrient composition of cow colostrum, we decided to conduct this experiment. The aim of this study was to determine the effect of grape pomace feeding on the nutritional quality and fatty acid composition of cow colostrum.

## 2. Materials and Methods

Slovak spotted cows, belonging to Simental breeds, were used in this experiment. Grape pomace (Pinot Gris variety) was obtained from the Academic Winery of the Slovak University of Agriculture in Nitra, located in the village of Oponice, Slovakia (GPS 48°27′31.8″N 18°09′44.5″E). The obtained grape pomace was a by-product of wine production and contained the skin, seeds, and part of the pulp. The fresh grape pomace was pre-dried for 4 days at 55 ± 5°C and stored in paper bags until the beginning of the experiment. Before addition to the diet of the pregnant cows in the GP group, the dried grape pomace was milled to a powder with a particle size of less than 1 mm using laboratory mills. The nutritional characteristic of the dried grape pomace used in this experiment was previously published in the articles of Kolláthová et al. [13,14] and Vašeková et al. [15]. In short, total polyphenols were equal to 27.38 mg GAE/g (GAE—equivalent gallic acid), total phenolic acids were equal to 13.27 mg CAE/g (CAE—equivalent caffeic acid), total flavonoids were equal to 0.12 mg QE/g (QE—equivalent quercetin), and antiradical activity DDPH was 9.17 mg TEAC/g (DPPH—2,2-diphenyl-1-picrylhydrazyl, TEAC—trolox equivalent antioxidant capacity); these quantities were determined from the bioactive compounds of the dried grape pomace. The dried grape pomace was composed 92.8% of dry matter (DM), 88.9% DM of organic matter, 9.4% DM of crude protein, 25.0% DM of acid detergent fibre, 28.4% DM of neutral detergent fibre, 0.35% DM of calcium, and 0.24% DM of phosphorus. The highest proportion of dried grape pomace fat was found as PUFA (69.13 g/100 g of fatty acids), followed by MUFA (16.69 g/100 g of fatty acids), and SFA (12.61 g/100 g of fatty acids). Animal care during this experiment was in accordance with national directive no. 377/2012 concerning the protection of animals used for scientific or educational purposes. The cows were under the care of a veterinarian throughout the experiment.

### 2.1. Animals and Experimental Design

The experiment was conducted on a commercial farm that breeds Slovak spotted cattle (PD Kozárovce, Slovakia, 48°18′58.32″N, 18°31′22.08″E ). Dry cows on this farm are housed in a loose system, with indoor straw bedding. The pens in this experiment were 24 × 4 m in dimension, for 8 cows. Feeding was performed once a day during the morning. This was followed by continual feed pushing throughout the day. The following selection criteria were used for the cows: the same breed—Slovak spotted cattle; similar calculated day of calving; and the number of lactations of the selected cows was in accordance with the number of lactations of the whole herd. The average lactation number of dry cows in the control and experimental group was 2.38. The average milk yield of cows in lactation before conducting the experiment was 8066 kg. A total of 16 Slovak spotted dry cows in last week of pregnancy were randomly divided into two groups (8 per group): CON = basal diet for dry cows in the last week of pregnancy; and GP = the same diet as in the CON group, with the addition of 0.116 kg/cow/day of the grape pomace powder (as topdressing on the diet). The composition of the diet given to the dry cows in the last week of pregnancy according to groups, and the diet given to the cows after calving from both groups is shown in Table 1. The amount of grape pomace powder (0.116 kg/cow/day) in the GP group was set according to results of our previous study with ruminants [16,17]. The calculated dietary intake per cow/day was 22.5 kg of fresh matter in the CON group and 22.62 kg of fresh matter in the GP group. The calculated dietary intake after calving was 43.25 kg of fresh matter per cow/day in both groups. During the entire experiment, water was provided ad libitum. There was 1 cow waterer per 8 cow group.

### 2.2. Colostrum Sample Collection and Storage

Within 30 min after calving, cows were milked with a vacuum milking machine. After milking and mixing, the first colostrum sample (0 h) was taken. All other colostrum samples were taken in a similar manner following this time schedule: 12th hour (12 h), 24th hour (24 h), 36th hour (36 h), 48th hour (48 h), and 60th hour (60 h) after the first colostrum sampling. In total, 100 mL of colostrum were taken into plastic tube, stored in a portable fridge (+ 7 °C), and analysed on the day of sampling.

### 2.3. Laboratory Analysis

On day of sampling, all the colostrum samples were transported to the National Reference Laboratory for Milk and Milk Products, a detached testing laboratory in Lužianky (State Veterinary and Food Institute, Slovakia). The nutritional composition of the colostrum (solids, fat, protein, lactose, ash, casein, and solid non-fat) and the fatty acid concentrations (sum of PUFA, sum of MUFA, sum of SFA, palmitic acid C16:0, stearic acid C18:0, oleic acid C18:1, sum of omega-6 fatty acids (n-6 FA), and sum of n-3 FA) were determined by DairySpec FT (Bentley Instruments, Chaska, MN, USA), according to the manufacturer’s instructions. The somatic cell count was analysed using a Fossomatic 500 (Foss, Denmark) (thousands of somatic cells per millilitre). The specific gravity of colostrum was determined with a colostrum densimeter (Kruuse, Sherburn in Elmet, UK) immediately after mixing of the obtained colostrum. The nutritional characteristics of the diets were analysed at the Laboratory of Quality and Nutritional Value of Feeds (Slovak University of Agriculture in Nitra, Slovakia), according to standard laboratory methods [18]. In brief, dry matter was determined gravimetrically after drying the sample at (103 ± 2 °C). Crude protein was evaluated as the total nitrogen content, as determined by the Kjeldahl method (Nx6.25). Ether extract was determined by extraction and the gravimetric method according to the Soxhlet principle without previous acid treatment. Crude fibre content was determined gravimetrically as the difference between residues after hydrolysis and after combustion. Ash was determined by measuring the resulting inorganic residue weight after ignition in a Muffle furnace at 550 °C. Nitrogen-free extract (NFE) was calculated according to the formula NFE = dry matter − (crude protein + crude fat + crude fibre + ash). Starch was determined by the polarimetric method, and sugar was measured according to the Luff–Schoorl method. Acid detergent fibre after hydrolysis in acid detergent soluble of cetaltrimetylamoniumbromid and sulphuric acid. Neutral detergent fibre content was determined in the Ankom 200 Fiber Analyzer (Ankom Technology, New York, NY, USA) according to procedures derived from Van Soest et al. [19]. The concentration of net energy for lactation was calculated according to the formula published by Sommer [20].

### 2.4. Statistical Analysis

For the statistical analyses and to maintain a normal distribution of data, the somatic cell count (SCC) was converted into a somatic cell score (SCS) using the following formula: SCS = log2 (SCC/100) + 3 [21]. The obtained data were statistically processed in IBM SPSS v. 26.0. The mean and standard deviation were generated using one-way ANOVA. The statistical significance of the differences in the determined parameters between groups (CON versus GP) was determined using *t*-tests. Orthogonal comparisons were conducted using a multivariate general linear model with hour and group as fixed factors (y = i + h + g + h*g, where y = colostrum nutrient, i = intercept, h = hour, and g = group) to determine the linear effects at 0, 12, 24, 36, 48, and 60 h from calving. A *p*-value less than 0.05 was considered to be significant.

## 3. Results

The concentration of base nutrients and fatty acid composition of the colostrum is shown in Table 2 and Table 3. In both groups, between the first udder secretion after calving and colostrum sampled at the 60th hour after calving, the concentrations of solids, protein, casein, ash, and solid non-fat decreased, whereas lactose increased linearly (*p* < 0.001) (Table 2). The concentration of fat increased during the first 24 h after calving and decreased from 24 h to 48 h. The specific gravity value decreased up to the 36th hour after calving and then remained the same. Within all sampling times, all differences between the CON and GP groups were insignificant (*p* > 0.05), except SCS at 12 h.

SFA was the group of colostral fatty acids with the highest concentration in both groups and for all the sampling times. PUFA were found in the lowest concentration in the cow colostrum. For the first 60 h after calving, oleic acid (C18:1) was the most abundant single FA in the cow colostrum. The n-3 FA concentration was the highest in the first udder secretion in both groups (0 h). The highest concentration of n-6 FA was detected in the colostrum of cows in the GP group at 0 h. For all the sampling times, a non-significant effect (*p* > 0.05) was determined as regards the FA concentration as a result of feeding dried grape pomace during the last week of pregnancy (Table 3). PUFA (*p* < 0.05) linearly decreased and SFA (*p* < 0.001) linearly increased in both groups, whereas MUFA (*p* < 0.05) only linearly increased in the GP group (Table 3).

## 4. Discussion

In dairy cows, the production of milk nutrients is affected by genetics and nutrition [22,23]. This is also true for colostrum. The protein concentration from the colostrum sampled immediately after calving from dry cows fed with additional grape pomace was similar to that found in the control group (15.2 versus 13.4%). These values are higher than those published by Puppel et al. [24] in their review article (13.3%). Because there is little information about the effect of grape pomace feeding on cow colostrum, the results are discussed in relation to articles published on the effect of grape pomace feeding on cow milk.

Scuderi et al. [7] did not find a higher concentration of milk proteins in cows fed additional grape marc; however, they found a higher concentration of immunomodulatory proteins, including bioactive proteins, which could be a feasible method to enhance the nutritional value of milk or, in this case, of colostrum. The results of a feeding experiment concerning the effect of grape pomace on cow milk parameters were published Nistor et al. [25]. Similar to this study, they found insignificant differences in milk protein concentrations between groups. Moreover, long-term grape pomace supplementation in dairy cows did not exhibit an effect on the concentration of milk protein [26]. Nielsen and Hansen [27] provided 4.5 g of grape pomace per cow per day and concluded that the inclusion of a small amount of grape pomace as a feed additive does not increase protein yield. Ianni et al. [9] concluded that dietary enrichment with grape pomace did not affect the milk composition. Gessner et al. [28] reported an increased milk yield and increased daily milk protein yield after feeding with grape seed and grape marc meal extract. They speculated that these increases could be due to a reduced ruminal degradation of crude protein from the diet, leading to an increased amount of protein available in the small intestine. Their findings are supported by the results of Moate et al. [29] and Ishida et al. [30], who reported that feeding with polyphenol-rich grape products increased milk yield by increasing the flux of protein into the small intestine. Nevertheless, in this study, we did not find the addition of grape pomace to have a positive or negative effect on the concentration of colostral proteins.

Sato et al. [31] reported that cow colostrum is fairly constant in terms of total ash content and specific gravity, which is not in accordance with the result of this study. Sato et al. [31] also concluded that the percentages of total proteins, fat, and lactose in the colostrum fluctuate widely. Similar to the findings of Nardone et al. [32], from 0 h to 36 h after calving, an increase in lactose concentration was detected in both groups of cows. The lowest concentration of lactose in colostrum at 0 h was followed by an increase over time. Zabielski et al. [33] stated that the concentration of lactase in the neonatal calf is lowest at birth and increases over time. Similar to this study, Pauletto et al. [26] and Ianni et al. [9] did not find any effects related to grape pomace feeding on milk lactose concentration. However, Chedea et al. [12] found an increased (+15%) concentration of milk lactose from cows fed a diet containing dried grape pomace. Comparable to the results of this study, Godden [34] reported a lower concentration of ash in cow colostrum (1.11 and 1.18%). The lower concentration of lactose and the higher concentration of ash in comparison to other studies can be explained by the osmotic function of lactose in milk. Holt and Jenness [35] stated that milk with a low level of lactose has an elevated level of inorganic elements. The specific gravity of the first udder secretion after calving was in the range 1048–1072 g/L; this decreased over the time to an average specific gravity of milk of 1029 g/L [36,37,38]. The decrease in the specific gravity negatively correlates with the lactose concentration [36]. The somatic cell count is mainly affected by the health status and udder infection status of cows [39]. However, in general, the somatic cell count of colostrum is higher as compared to milk. Nguyen and Neville [40] attributed the high somatic cell count in colostrum to a physiological feature: the penetration of cells through leaky tight junctions between the mammary epithelial cells, rather than mastitic infection. In a review of McGrath et al. [38], a somatic cell score of cow colostrum between 3.70 and 4.15 was reported, which is slightly higher as compared to the results of this study. A lower value of SCS in the colostrum of cows from the GP group at 12 h (Table 2) can be explained by the effects of polyphenols from the grape pomace on the immune system, their anti-microbial effects, and the anti-inflammatory mechanisms of polyphenols [41,42,43]. On the other hand, Nielsen and Hansen [27] found grape pomace feeding to have no effect on the somatic cell count of milk.

A comprehensive description of the roles that colostral fat plays for newborn calves was provided by Contarini et al. [2]. Colostral fat supplies essential nutrients, which provide energy, increase metabolism, and protect the newborn against microbial infection. In addition, fatty acid oxidation is useful to promote active gluconeogenesis in order to maintain glucose homeostasis [44]. The concentration of fat in colostrum varies greatly. A concentration of 8.50% colostral fat at calving was published by Yaylak et al. [45] for Simmental cows, which is much higher as compared to the results of this study (4.0 and 4.8%). Scuderi et al. [7] investigated the effect of the inclusion of the grape marc in dairy cattle rations and, similar to this study, no effect on milk fat concentration was observed. Moreover, Chedea et al. [12] determined no significant difference in milk fat concentration after feeding with grape pomace. It must be noted that grape pomace feeding has a tendency toward a reduction in milk fat [12], which is comparable to the results of this study.

In this study, the time between colostrum sampling was shorter (12 h) than that in other studies. However, the evolution of fatty acid concentration is comparable to that reported by Contarini et al. [2]. Between 0 h and 60 h after calving, the concentration of SFA and MUFA increased and PUFA decreased (Table 3). No significant differences in colostral PUFA were detected in this study, which is in disagreement with the conclusions of Ianni and Martino [8]. They reported that ingestion of grape pomace increased the concentration of PUFA in the milk of dairy cows. Furthermore, Correddu [11] demonstrated the ability of polyphenols to decrease SFA and increase MUFA and PUFA in milk. A similar finding was published by Castellani et al. [10]. They concluded that supplementing dairy cow diets with grape pomace may enhance the milk’s fatty acid composition. Resconi et al. [46] reported that this change in SFA, MUFA, and PUFA in the milk of ruminants fed grape pomace could be caused by the content of linoleic acid and polyphenols in grape pomace, which could modulate the ruminal biohydrogenation of PUFA. In addition, grape residues also provide higher levels of non-fermentable fibre, and lignin, which could affect the ruminal digestibility, resulting in a decreased percentage of de novo fatty acids in milk [29,47]. On the other hand, Leiber et al. [48] stated that a certain level of PUFA in the colostrum is maintained by the animal, regardless of diet. The results of this study demonstrate that grape pomace intake at 0.116 kg/dry cow/day had no effect on colostrum fatty acid composition.

## 5. Conclusions

The additional of 0.116 kg/cow/day of dried grape pomace into the diet of dry cows had no effect on the analysed colostral nutrient or fatty acid parameters. Thus, grape pomace can be used as a nutrient source for dry cows.

## Figures and Tables

**Table 1:** The composition and nutritional characteristics of the diets provided for the 7 days before calving and after calving.

Group	The 7 Days Before Calving		After Calving
	CON	GP	CON and GP
**Ingredients of the Diet (%)**			
Maize silage	57.77	57.47	48.60
Alfalfa silage	17.77	17.69	25.40
Wheat straw	6.67	6.63	0.60
Rapeseed meal	5.33	5.31	5.50
Soy bean meal	3.56	3.54	3.50
Barley grain	3.56	3.54	2.30
Maize grain	1.78	1.77	5.80
Wheat grain	1.78	1.77	5.20
Oat grain	1.07	1.06	
Beet molasses			2.30
Mineral premix “dry cow” ^1^	0.71	0.71	
Mineral premix “lactating cow” ^2^			0.80
Grape pomace powder		0.51	
**Nutritional Characteristic**			
DM (%)	48.72	49.09	50.85
Crude protein (g/kg DM)	136.82	142.05	137.70
Ether extract (g/kg DM)	23.23	22.39	23.74
Crude fibre (g/kg DM)	213.94	219.94	182.05
NFE (g/kg DM)	542.80	534.01	582.31
Ash (g/kg DM)	83.21	81.61	74.20
Starch (g/kg DM)	222.31	219.15	252.21
Sugar (g/kg DM)	24.40	23.45	64.29
ADF (g/kg DM)	257.61	270.20	206.82
NDF (g/kg DM)	378.80	383.97	325.50
NEL (MJ/kg DM)	6.65	6.56	7.18
pH	4.70	4.71	4.80

CON—control group, GP—grape pomace group, DM—dry matter, NFE—nitrogen free extract, ADF—acid detergent fibre, NDF—neutral detergent fibre, NEL—net energy for lactation, ^1^—1 kg of mineral premix contains 4.0% Ca, 3.0% P, 4.0% Na, 10.0% Mg, 2500 mg Cu, 10,000 mg Zn, 7000 mg Mn, 50 mg Co, 200 mg I, 23 mg Se, 750,000 U.I. Vitamin A, 150,000 U.I. Vitamin D3, 7000 mg Vitamin E, 140 mg Biotin, 25,000 mg Niacinamid (Mikrop Čebín, Czech Republic), ^2^—1 kg of mineral premix contains 18.0% Ca, 2.5% P, 9.0% Na, 8.0% Mg, 2000 mg Cu, 5000 mg Zn, 4500 mg Mn, 25 mg Co, 120 mg I, 35 mg Se, 700,000 U.I. Vitamin A, 180,000 U.I. Vitamin D3, 3000 mg Vitamin E, 80 mg Biotin (Mikrop Čebín, Czech Republic).

**Table 2:** The characteristics of cow colostrum during the experiment (%).

Hour After Calving	0 h		12 h		24 h		36 h		48 h		60 h		Effect of Time ^†^	
Group	CON	GP	CON	GP	CON	GP	CON	GP	CON	GP	CON	GP	CON	GP
Solids x	22.1	23.0	19.1	18.8	16.6	15.6	15.7	14.7	14.4	14.0	14.2	14.6	+++	+++
SD	2.77	1.96	2.94	4.64	3.73	2.04	3.86	1.72	0.93	1.78	1.36	1.53		
Fat x	4.8	4.0	5.3	4.4	6.1	4.5	5.9	4.5	4.6	4.1	5.0	4.7		
SD	1.98	1.36	1.65	2.89	4.92	1.44	4.40	1.25	0.89	1.44	1.46	1.69		
Protein x	13.4	15.2	9.4	10.6	5.7	6.4	4.9	5.2	4.7	4.8	4.4	4.7	+++	+++
SD	1.75	2.12	2.85	2.04	1.67	1.08	0.59	0.57	0.37	0.37	0.21	0.30		
Lactose x	2.2	2.1	3.0	2.5	4.0	3.7	4.1	4.1	4.3	4.2	3.9	4.4	+++	+++
SD	0.50	0.73	0.84	0.69	0.35	0.52	0.30	0.29	0.15	0.28	0.79	0.25		
Ash x	1.7	1.7	1.4	1.4	0.9	1.0	0.8	0.9	0.8	0.8	0.9	0.9	+++	+++
SD	0.27	0.23	0.30	0.30	0.23	0.15	0.07	0.15	0.06	0.18	0.17	0.14		
Casein x	12.1	13.9	8.3	9.4	4.7	5.5	4.0	4.3	3.9	4.0	3.6	3.8	+++	+++
SD	1.74	2.08	2.67	1.88	1.88	0.96	0.77	0.50	0.31	0.35	0.15	0.27		
SNF x	16.2	17.8	13.1	13.7	10.4	10.8	9.8	10.0	9.7	9.8	9.2	9.8	+++	+++
SD	1.34	1.75	2.04	2.12	1.47	0.91	0.74	0.47	0.37	0.23	0.50	0.35		
SG x	1048	1047	1040	1039	1036	1035	1035	1035	1035	1035	1035	1035	+++	+++
SD	4	9	6	7	2	0	0	0	0	0	0	0		
SCS x	3.8	3.3	4.2	2.6 *	3.3	3.5	3.7	3.3	3.3	3.5	3.5	3.2		
SD	0.48	1.07	1.25	0.16	0.90	1.25	1.48	1.43	1.12	1.76	1.34	1.33		

CON—control group of cows (*n* = 8), GP—group of cows (*n* = 8) feed during last week of pregnancy with addition of dried grape pomace, SNF—solid-non-fat, SG-specific gravity (g/L), SCS—somatic cell score, x‚—mean, SD—standard deviation, *—GP value differs significantly from the control group at a similar time point, ^†^—linear effect of graded time from calving on nutrient concentration when +++ *p* < 0.001.

**Table 3:** The concentrations of fatty acids in the cow colostrum (g/100 g fat).

Hour After Calving	0 h		12 h		24 h		36 h		48 h		60 h		Effect of Time ^†^	
Group	CON	GP	CON	GP	CON	GP	CON	GP	CON	GP	CON	GP	CON	GP
PUFA x	11.3	11.0	7.3	8.1	7.5	5.0	7.1	5.2	6.3	6.2	7.3	6.5	+	+
SD	5.29	3.75	3.32	3.23	4.76	1.74	3.52	1.23	1.88	1.70	2.62	1.34		
MUFA x	30.0	26.7	33.7	30.4	34.1	33.6	35.4	33.8	33.8	34.9	33.4	35.1		+
SD	5.26	10.64	4.08	6.45	3.74	3.66	5.74	3.92	6.17	5.50	8.74	3.71		
SFA x	44.7	38.2	52.4	44.8	58.1	55.2	60.1	59.0	61.7	56.4	62.8	60.0	+++	+++
SD	14.51	12.44	7.40	12.25	12.63	9.70	8.61	6.41	5.58	8.40	4.49	4.00		
PA x	24.9	21.6	23.7	21.8	19.3	21.7	19.4	21.6	22.3	18.2	22.6	20.3		
SD	5.62	7.68	3.83	4.43	5.24	3.42	6.07	3.91	4.60	7.63	2.75	3.92		
SA x	4.0	3.3	6.1	4.0	7.6	7.6	8.8	8.8	9.2	9.0	9.5	9.7	+++	+++
SD	0.87	4.50	2.19	2.44	1.53	1.62	1.14	0.95	1.47	2.03	2.17	1.73		
OA x	35.8	38.5	33.7	35.5	31.7	32.3	31.8	30.3	29.6	31.6	28.9	31.1	+	++
SD	8.92	7.34	5.60	4.62	5.65	6.04	7.04	4.93	7.34	5.71	8.51	3.63		
n-6 FA x	2.5	4.7	1.2	2.4	2.3	1.6	2.6	2.2	2.9	2.5	3.5	3.0	++	
SD	2.19	5.63	0.76	2.74	1.72	0.74	1.12	0.71	0.51	0.63	0.54	0.59		
n-3 FA x	1.6	1.5	1.3	1.3	1.4	1.2	1.4	1.1	1.4	1.3	1.4	1.4		
SD	0.49	0.40	0.26	0.21	0.33	0.16	0.14	0.12	0.18	0.15	0.17	0.23		
n6/n3 x	1.4	3.5	0.9	2.1	1.6	1.4	1.8	1.8	2.1	1.9	2.4	2.3	+++	
SD	1.15	5.15	0.51	2.81	1.11	0.64	0.75	0.59	0.32	0.60	0.38	0.56		

CON—control group of cows (*n* = 8), GP—group of cows (*n* = 8) fed during last week of pregnancy with the addition of dried grape pomace, PUFA—polyunsaturated fatty acids, MUFA—monounsaturated fatty acids, SFA—saturated fatty acids, PA—palmitic acid (C16:0), SA—stearic acid (C18:0), OA—oleic acid (C18:1), n-6 FA—omega-6 fatty acids, n-3 FA—omega-3 fatty acids, n-6 FA/n-3 FA ratio, x—mean, SD—standard deviation, at a similar time point, no significant difference between groups was found, ^†^—linear effect of graded time from calving on fatty acids concentration when +++ *p* < 0.001, ++ *p* < 0.01, + *p* < 0.05.

## Data Availability

The data presented in this study are available on request from the corresponding author.
